# Could the Inhibition of Endo-Lysosomal Two-Pore Channels (TPCs) by the Natural Flavonoid Naringenin Represent an Option to Fight SARS-CoV-2 Infection?

**DOI:** 10.3389/fmicb.2020.00970

**Published:** 2020-04-30

**Authors:** Antonio Filippini, Antonella D'Amore, Fioretta Palombi, Armando Carpaneto

**Affiliations:** ^1^Unit of Histology and Medical Embryology, Department of Anatomy, Histology, Forensic Medicine and Orthopaedics, SAPIENZA University of Rome, Rome, Italy; ^2^Department of Earth, Environment and Life Sciences (DISTAV), University of Genoa, Genoa, Italy; ^3^Institute of Biophysics, National Research Council, Genoa, Italy

**Keywords:** Naringenin, SARS - CoV, antiviral treatment, endo-lysosomal dysfunction, therapeutic targets, two-pore channel (TPC)

In the present opinion article we highlight evidence from different laboratories to drive the attention of the scientific community on the role played by endo-lysosomal Two-Pore Channels (TPCs) in viral infection. In particular, cross linking our recent data and existing literature, we focus on evidence indicating that virus intracellular pathway could be targeted by a novel occurring TPCs inhibitor, the flavonoid Naringenin. A conceptual framework is presented for considering such a strategy as a promising approach to limit the infection mediated by the novel coronavirus SARS-CoV-2. Our hypothesis offers a perspective on a novel molecular target, TPCs, which could be exploited for a pharmacological blockade of SARS-CoV-2 infectivity.

The Coronaviruses are emerging viruses that are able to cross the species barrier and cause severe diseases in humans. Two such recent events are the highly pathogenic Serious Acute Respiratory Syndrome-related CoV (SARS-CoV) that became apparent in Southern China in 2003 and Middle East Respiratory Syndrome-related CoV (MERS-CoV), which emerged in 2012.

In the present dramatic outbreak of coronavirus disease-19 (COVID-19) that is caused by SARS-CoV-2 [recently reviewed in (Lai et al., 2020)], while science and medicine are striving to develop efficient treatments, we urge researchers to take into serious consideration a novel pharmacological strategy, highly promising for efficient and safe prophylaxis and therapy. What we recommend is to focus on the role played by the endo-lysosomal two-pore channel family (TPCs) in viral infection and on the feasibility of blocking the intracellular pathway of the virus by inhibiting these channels. Cross-analysis of data published over different times, experimental models and approaches gives direct and indirect evidence in support of this proposal.

First of all, Sakurai et al. (2015) demonstrated that TPC2 is required for release of the Ebola viral genome into the host cell during Ebola virus entry pathway and, interestingly, TPC2 inhibitors such as tetrandrine have proven capable of blocking virus trafficking and prevented infection *in vitro* and in mice *in vivo*. Intriguingly, our recent evidence has shown that the activity of human TPC channels can be inhibited by a natural flavonoid compound, in fact present in citruses and tomatoes, Naringenin (Pafumi et al., 2017). In our opinion this evidence gives priority to Naringenin (Nar) for testing as a safe potential weapon against the present infection. The rationale for a defense line based on inhibiting lysosomal pro-viral activity through TPC2 inhibition is further supported by the following direct and indirect data. It has been shown (Gunaratne et al., 2018) that knockdown and pharmacological inhibitors of both TPC2, mainly expressed in late endosomes/lysosomes, and TPC1, which mainly localizes to early endosomes, attenuate intracellular trafficking of coronavirus MERS-CoV through the endolysosomal system, even though the data were obtained using an artificial virion. Besides TPC2, Nar is also an inhibitor of TPC1 activity with an IC50 of about 500 μM therefore larger than for TPC2 (about 200 μM) (Pafumi et al., 2017).

Relevant and very recent *in vitro* evidence has shed light to the efficacy of chloroquine to fight SARS-CoV-2 infection through lysosomal alkalinization (Touret and de Lamballerie, 2020; Wang et al., 2020). As a matter of fact, chloroquine acts as a weak base and accumulates in the lysosomes quenching their acidic pH, thereby halting autophagic degradative flux (Homewood et al., 1972). In line with this evidence, interestingly, it has been found that loss of TPC2 leads to an increase in melanosome/lysosome pH (Cang et al., 2013; Ambrosio et al., 2016; Bellono et al., 2016). In fact, TPC2 was shown to be involved in the control of human melanosome luminal pH: actually in TPC2-KO human melanotic MNT-1 cells, and in primary melanocytes subjected to TPC2 knockout by the CRISPR/Cas9 gene editing system, the lumen of melanosomes is more alkaline than in control cells (Ambrosio et al., 2016). Bellono et al. (2016) also hypothesized that TPC2 can regulate melanosome pH producing a cation counterflux to enhance V-ATPase H^+^ transport into the melanosome lumen, consistent with the requirement for an inward cation current in lysosomal acidification (Steinberg et al., 2010). In addition, Cang et al. (2013) demonstrated a shift toward alkalinization in TPC2^−/−^ macrophage lysosomes after starvation.

Since viral replication takes place in specific cellular compartments induced by viral proteins which modify cell organelles to create sites for replication, hidden from innate immunity, membrane fusion mechanisms are crucial events in the infection process. To this purpose, the virus S protein consists of two subunits, S1 and S2, with S1 providing the receptor binding function through the entry receptor ACE2 and S2 providing fusion activity. Interestingly, the subunits are cleaved from the complete S by host cell proteases (cysteine proteases cathepsin B and L, furin proteases and cellular serine protease TMPRSS2) and, following receptor binding by S1, the fusion mechanism of S2 acts to bring the viral and cellular vesicles membranes into such close proximity that fusion occurs (reviewed in Alsaadi and Jones, 2019). In this context, it should be noted that the opening of TPCs induces a strong sodium-driven depolarization in the endo-lysosomal membrane (Wang et al., 2012; Boccaccio et al., 2014; Cang et al., 2014; Lagostena et al., 2017), which is supposed to enhance membrane fusion mechanisms (Wang et al., 2012). In line with this hypothesis, COS-1 cells transfected with human TPC2 have larger lysosomes than cells transfected with a non-functional form of the channel. Moreover, it was recently shown (Freeman et al., 2020) that TPCs are directly involved in sodium efflux, which, in parallel with chloride movement, regulates osmolyte release in endocytic vacuoles, with significant modification of vacuolar surface-to-volume ratio. Therefore, inhibition of TPCs should both impair the fusogenic potential of the endo-lysosomal system and alter the normal trafficking, which, in turn, could be a limit for viral replication (Alsaadi and Jones, 2019). Very recently, unique features of TPC2 in the response to different agonists have been published (Gerndt et al., 2020) expanding the characterization of this channel, hence the range of potential approaches to pharmacologically control the intracellular pathway of the virus.

The use of Nar, one of the main flavonoids present in the human diet, as a specific inhibitor of TPCs (Benkerrou et al., 2019) has several advantages. Nar is a hydrophobic molecule able to cross biological membranes and to reach the intracellular compartments (endosomes and lysosomes) where TPCs are localized. The toxicity of Nar is low: concentrations greater than 1 mM do not affect human hepatocytes viability (Nahmias et al., 2008) and, in mice, doses up to 1,500 mg/kg given by intraperitoneal injection did not induce marked elevation of liver enzymes or cause animal death (Nahmias et al., 2008). Interestingly, in the same study (Nahmias et al., 2008), Nar was shown to be effective to reduce Hepatitis C virus secretion by 80% when added at 200 μM in infected Huh7.5.1 human hepatoma cell line. Moreover, that Nar treatment could be a promising strategy to inhibit virus replication and infection is further confirmed by interesting studies on the influenza A virus, dengue virus and Zika virus (Dong et al., 2015; Frabasile et al., 2017; Cataneo et al., 2019). Antiviral effect of some flavonoids and Nar through blocking viral proteases activity in different experimental models has been also reported (de Sousa et al., 2015; Lulu et al., 2016; Lim et al., 2017; Jo et al., 2020). Of note, Nar has been shown to ameliorate acute inflammation (Jin et al., 2017) as well as lung fibrosis (Zhang et al., 2018), which could represent a therapeutic advantage. In particular, Zeng et al. demonstrated that Nar suppresses inflammatory cytokine production through both transcriptional and post-transcriptional mechanisms (by regulating lysosome function) resulting in the inhibition of TNF-α and IL-6 secretion by macrophages and T cells (Jin et al., 2017; Zeng et al., 2018). Clinical trials analyzing the therapeutic potential of Nar have been recently reviewed (Salehi et al., 2019) and an important clinical trial on the pharmacokinetics and metabolism of Nar has just been reported, indicating the strong interest around this compound (Bai et al., 2020). While this manuscript was under review, an article by Ou et al. (2020) demonstrated that TPC2 is a key player for SARS-CoV-2 entry in 293/hACE2 cells, consistent with our findings and further supporting our hypothesis.

In conclusion, these considerations offer a perspective on specific molecular targets, TPCs, and underpin a role for Naringenin as pharmacological blockade of SARS-CoV-2 infectivity providing further support for exploration of TPCs inhibition as novel antiviral therapy.

## Data Availability Statement

The raw data supporting the conclusions of this article will be made available by the authors, without undue reservation, to any qualified researcher.

## Author Contributions

AF and FP conceived the hypothesis and analyzed the data. AF and AC shared the study. AF, AD'A, FP, and AC designed the conceptual framing and wrote the manuscript.

## Conflict of Interest

The authors declare that the research was conducted in the absence of any commercial or financial relationships that could be construed as a potential conflict of interest.
